# Multi-omics dissection of fatty acid metabolism heterogeneity identifies PRDX1 as a prognostic marker in bladder cancer

**DOI:** 10.3389/fimmu.2025.1669822

**Published:** 2025-09-11

**Authors:** Li Wang, Zhe Chang, Si-yu Chen, Jian-wei Yang, Kang-yu Wang, Kun-peng Li, Shun Wan, Shan hui Liu, Li Yang

**Affiliations:** ^1^ Department of Urology, Gansu Province Clinical Research Center for Urinary System Disease, The Second Hospital & Clinical Medical School, Lanzhou University, Lanzhou, China; ^2^ Institute of Urology, Gansu Province Clinical Research Center for Urinary System Disease, The Second Hospital & Clinical Medical School, Lanzhou University, Lanzhou, China

**Keywords:** fatty acid metabolism, BLCA, PRDX1, machine learning, urology

## Abstract

**Background:**

Fatty−acid metabolism (FAM) is rewired in bladder cancer (BLCA), yet its impact on intratumoral diversity and patient outcome is unclear.

**Methods:**

To characterize FAM heterogeneity, we integrated spatial and single-cell transcriptomic approaches. We employed high-dimensional weighted correlation network analysis (hdWGCNA) alongside five distinct enrichment methods (ssGSEA, AddModuleScore, AUCell, singscore, and UCell) to identify modules with elevated FAM activity. Subsequently, machine learning algorithms were applied to bulk RNA sequencing datasets to pinpoint the key gene with highest predictive value. This candidate underwent validation through functional experiments and analysis of clinical specimens.

**Results:**

Malignant epithelial cells displayed the strongest FAM activity. Cross−platform scoring and co−expression analysis produced a refined high−FAM gene set. Integrating this signature with bulk datasets singled out PRDX1 as a key driver. PRDX1 was up−regulated in tumors, predicted poorer prognosis, and was enriched in malignant epithelial cells. Silencing PRDX1 curtailed BLCA cell proliferation, migration, and invasion.

**Conclusions:**

PRDX1 emerges as a FAM−linked oncogenic biomarker that fosters BLCA progression. These findings define the metabolic hierarchy of BLCA and nominate PRDX1 as a candidate target for personalized therapy.

## 1 Introduction

Bladder cancer (BLCA) represents a major urological malignancy, accounting for roughly 573,000 newly diagnosed patients and 212,000 fatalities worldwide during 2020 (1, 2). Urothelial carcinoma comprises more than 90% of diagnoses in industrialized nations, where both geographic location and patient age significantly affect disease occurrence (3, 4). Even with progress in surgical techniques and systemic treatments, patient prognosis continues to be dismal owing to high rates of recurrence, disease advancement, and therapeutic resistance, especially among those with muscle-invasive or metastatic tumors (5, 6). Recurrence affects around 50-70% of non-muscle-invasive tumors, while muscle invasion develops in as many as 30% of patients (7). Patients with metastatic disease experience survival rates below 10% at five years (8, 9). Such statistics underscore the urgent requirement to identify molecular mechanisms underlying tumor aggressiveness and create superior treatment approaches.

The reprogramming of cellular metabolism is a hallmark of BLCA, with lipid alterations playing a central role in tumor initiation and progression (10). Aberrant fatty acid metabolism (FAM) in BLCA is not merely a byproduct of malignancy but actively supports tumor growth, survival, and adaptation (11). Specifically, BLCA cells display enhanced fatty acid uptake and synthesis, reduced fatty acid oxidation (FAO), and marked lipid droplet accumulation (12), changes that promote oxidative stress resistance and membrane stability under hypoxic or nutrient-limited conditions (13). Moreover, altered lipid composition has been linked to epithelial-to-mesenchymal transition (EMT), invasion, and immune evasion in aggressive BLCA subtypes (14).

Although PRDX1 has been reported as an oncogenic factor and adverse prognostic marker in BLCA, its role has not previously been examined in the context of FAM heterogeneity. By integrating multi-omics data and machine learning, our study uniquely positions PRDX1 as a FAM-linked biomarker, thereby uncovering a novel layer of its functional relevance in BLCA.

Traditional bulk transcriptomic methods lack cellular resolution, limiting insights into cell-specific metabolic reprogramming. Single-cell RNA sequencing (scRNA-seq) addresses this by uncovering transcriptional heterogeneity and rare cell populations, offering new perspectives on FAM dynamics. Spatial transcriptomics (ST) further complements scRNA-seq by preserving tissue context and revealing spatial patterns of metabolic alterations. However, limitations in sample size and interpatient variability reduce the generalizability of current findings. Thus, integrating multi-omics data is essential to overcome these challenges, enabling more accurate identification of metabolic biomarkers and therapeutic targets in BLCA (15).

In this study, we integrated scRNA-seq, ST, and bulk RNA-seq data to systematically explore the functional landscape of FAM in BLCA. Single-cell analysis revealed pronounced heterogeneity in FAM activity among distinct cellular populations, with malignant urothelial cells exhibiting elevated FAM-related gene expression. Using a combination of machine learning approaches, we identified PRDX1 as a central regulator of FAM dysregulation. PRDX1 expression correlated with tumor aggressiveness and poor prognosis in public BLCA cohorts. Functional validation through *in vitro* assays confirmed that PRDX1 modulates key enzymes involved in lipid metabolism, thereby contributing to tumor cell proliferation and oxidative stress resistance. This study reveals novel metabolic features of BLCA and identifies PRDX1 as a viable target for intervention.

## 2 Methods

### 2.1 Data collection

**Figure 1:**
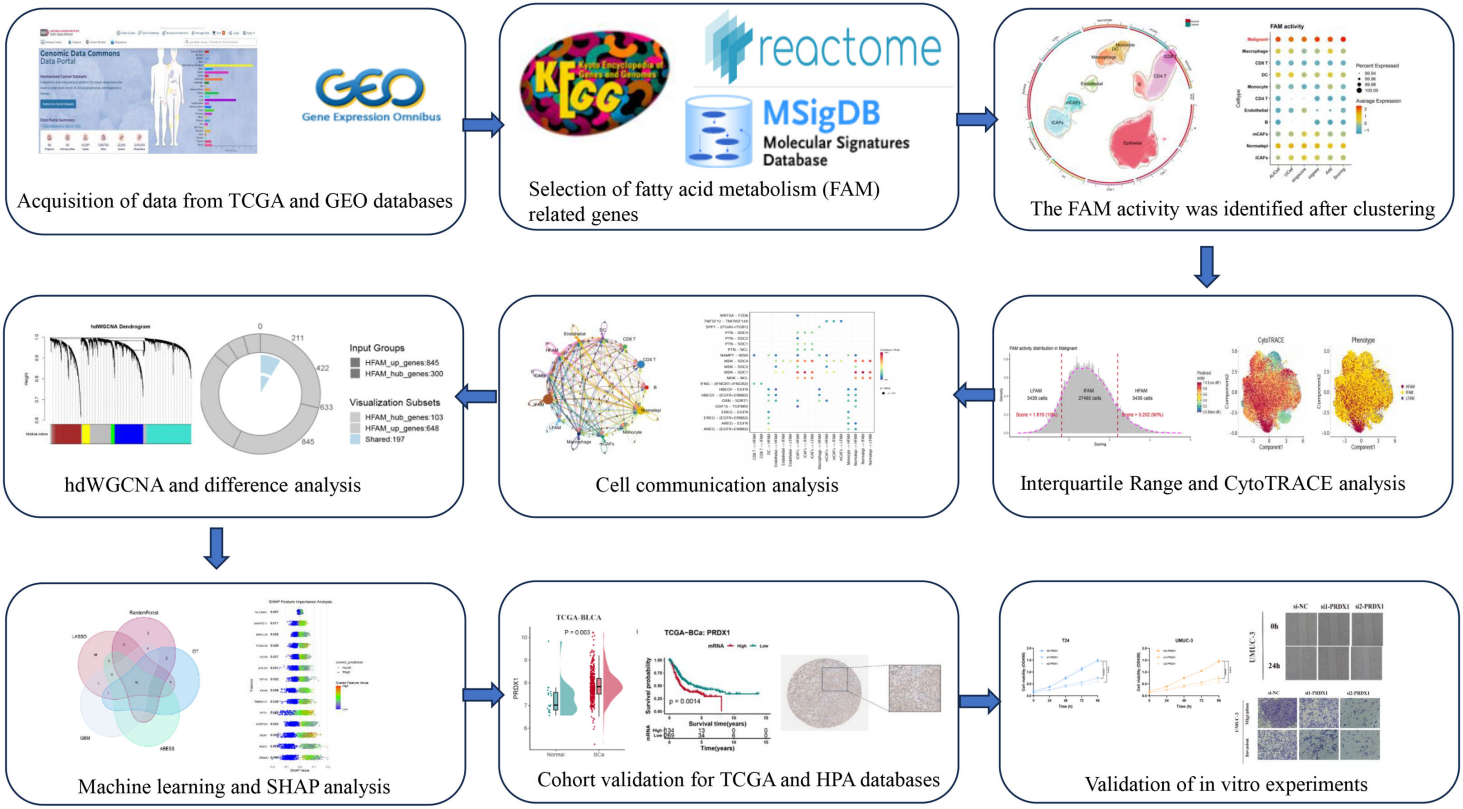
Work flow of the study.

From the GEO repository, we obtained seven single-cell RNA sequencing datasets (GSE129845, GSE130001, GSE135337, GSE146137, GSE190888, GSE192575, and GSE211388), which included 34 total samples consisting of 30 tumors and 4 normal tissue controls (16). Clinical information and bulk RNA sequencing data were acquired from both TCGA and GEO databases (GSE13507, GSE32984). To incorporate spatial gene expression information, we also utilized a spatial transcriptomics dataset (GSE171351). Proteomic profiles from urine samples were collected from five individuals with BLCA alongside five healthy participants, following methods described in previous work (17). Detailed information about all the datasets used in this study was provided in **Supplementary Table S1**. We assembled a collection of 323 fatty acid metabolism-related genes by integrating information from multiple sources: KEGG, REACTOME, MSigDB v5.2 Hallmark gene sets, and existing publications (18) (see **Supplementary Table S2**). Additionally, protein expression of the related genes was examined through the Human Protein Atlas (HPA) (https://www.proteinatlas.org/) database. The flow chart illustrating the operational procedure in this study (**Figure 1**).

### 2.2 Data analysis

Raw scRNA-seq data were processed into Seurat objects and integrated as previously described (19). Quality control retained high-quality cells based on the following thresholds: (1) 200–5,000 genes detected per cell; (2) ≤15% mitochondrial gene content; and (3) >3% erythrocyte gene expression. After normalization, a total of 115,628 qualified cells were included for downstream analysis. Batch effects were corrected using Harmony integration. The data underwent log-normalization followed by scaling through linear regression methods. Using the “FindVariableFeatures” function, we selected the 3,000 most variable genes. Principal component analysis (PCA) was employed for dimensionality reduction, with subsequent graph-based clustering executed through the “FindClusters” algorithm. UMAP was utilized for visualization, while canonical marker gene expression guided the annotation of distinct cell populations. For bulk RNA-seq data, raw counts were log2-transformed and normalized using the DESeq2 pipeline or limma-voom.

### 2.3 High-dimensional weighted correlation network analysis

Leveraging the hdWGCNA algorithm for high-dimensional weighted gene co-expression network analysis, we constructed cell-type-specific networks within high resistance activity state at single-cell resolution (20). Scale-free topology networks were implemented using a soft threshold power of 12, maintaining >0.85 model fit index. The Construct Network function identified robust gene modules, with eigengene connectivity (kME) quantifying module-associated expression profiles (21). Key disease-relevant modules were subsequently screened via UCell scoring (22). Shared candidate genes derived from hdWGCNA underwent differential expression validation, following standardized protocols documented in the official pipeline (https://smorabit.github.io/hdWGCNA/).

### 2.4 FAM score in scRNA

To assess FAM activity within single-cell RNA sequencing datasets, we employed five distinct computational approaches: AUCell, UCell, singscore, ssGSEA, and AddModuleScore (23, 24). Based on IQR range methods, we stratified malignant cells into three distinct FAM phenotypic categories: low FAM activity state (LFS), dynamic transition FAM activity state (DTFS), and high FAM activity state (HFS). The FindMarkers function enabled identification of genes showing differential expression in association with increased FAM activity.

### 2.5 Infer the malignant epithelial cells

The inferCNV algorithm was utilized to detect malignant epithelial cells through chromosomal copy number variation (CNV) assessment. By computing the average squared magnitude of CNV across all chromosomes, we generated a malignancy score that captured the extent of clonally expanded CNV alterations (25). Cell malignancy status was determined through bimodal distribution analysis of CNV scores relative to normal reference profiles, enabling statistically robust epithelial malignancy classification.

### 2.6 Cellular trajectory reconstruction analysis using gene counts and expression analysis

Cellular differentiation trajectories were inferred using CytoTRACE (26), a computational framework that quantifies developmental potential from single-cell transcriptomes. This algorithm employs an entropy-based metric calculated from two key features: (1) the number of expressed genes per cell, and (2) the distribution of highly variable genes associated with undifferentiated states. Malignant epithelial cells identified through prior CNV analysis were subjected to CytoTRACE scoring, with lower scores indicating advanced differentiation status and higher scores signifying primitive stem-like states.

### 2.7 Cell communication

Cell-cell communication dynamics were analyzed using CellChat (v1.6.0) with integrated ligand-receptor co-expression profiling (27). The standard pipeline employed CellChatDB-human containing 2,021 curated interactions. Cell-type-specific signaling networks were resolved through differential expression analysis of ligands/receptors across defined subpopulations, quantifying interaction probability alterations (28).

### 2.8 Screening of feature genes

To identify the most informative feature genes, we first applied six feature selection algorithms—Random Forest (RF), LASSO regression, Decision Tree (DT), Adaptive Best Subset Selection (ABESS), and Gradient Boosting Machine (GBM)—each of which offers distinct strengths for high-dimensional data analysis (29). The selected features were then used to build predictive models. Specifically, we evaluated eight classification algorithms, including k-nearest neighbor (KNN), linear discriminant analysis (LDA), logistic regression (LR), Naïve Bayes (NB), random forest (Ranger), recursive partitioning and regression trees (RPART), support vector machine (SVM), and extreme gradient boosting (XGBoost), and compared their performance to select the optimal model. To further interpret the contribution of selected features, we applied SHAP (SHapley Additive exPlanations) analysis. SHAP values provide a unified framework derived from game theory to quantify the impact of each gene on model predictions. In this context, positive SHAP values indicate that a gene promotes classification into the high-risk group, whereas negative values suggest association with the low-risk group. For biological validation, we examined the enrichment of the identified core genes across cell types using scRNA-seq data, and further confirmed their expression differences in TCGA-BLCA and bulk RNA-seq datasets with the Wilcoxon rank-sum test. Finally, the predictive performance of the model was assessed using the area under the receiver operating characteristic (ROC) curve.

### 2.9 Cell culture and transfection

BLCA cell lines (T24, UMUC-3, J82, and 5637) along with the normal urethral epithelial cell line SV-HUC-1 were obtained from the Gansu Province Clinical Research Center for Urinary System Diseases. SV-HUC-1 cells were grown in Ham’s F12K medium, while all malignant cell lines were cultivated in RPMI-1640 (Shanghai Yuanpei Biotechnology). Both culture media were supplemented with 10% fetal bovine serum (FBS) from PAN Biotech and 1% penicillin-streptomycin at 100 U/mL-100 μg/mL concentration from Solarbio. Cells were incubated under standard conditions maintaining 37 °C temperature, 5% CO_2_ atmosphere, and appropriate humidity levels. The small interfering RNAs (siRNAs) directed against PRDX1 were procured from Tsingke Biological, and the transfection reagent was sourced from Shanghai GenePharma Biotechnology. The efficacy of the knockdown was validated using Quantitative RT−PCR (qRT−PCR) analysis at 36 hours post-transfection. Moreover, concurrent phenotypic experiments were conducted using the same procedure.

### 2.10 Quantitative RT−PCR

Total RNA extraction from four BLCA cell lines (UMUC-3, J82, T24, and 253J) was performed using TRIzol reagent (Invitrogen, USA), followed by spectrophotometric quantification of RNA concentrations. One microgram of extracted RNA underwent reverse transcription to cDNA using the AJ reverse transcription kit. qRT-PCR analysis was performed using a BIO-RAD CFX-96 system, with gene expression levels determined through the 2^-^ΔΔCt method and normalized against β-actin. Data are presented as mean ± SD. Primer sequences are detailed in **Supplementary Table S3**.

### 2.11 Western blotting

Total protein was extracted using RIPA buffer (P0013B, Beyotime, China) supplemented with protease inhibitors. Protein concentrations were determined by the bicinchoninic acid (BCA) assay. Samples were separated by SDS-PAGE and transferred onto PVDF membranes. Membranes were blocked with 6% non-fat dry milk before incubation with primary antibodies overnight at 4 °C. Protein bands were identified utilizing the Odyssey imaging system in conjunction with the appropriate secondary antibody (926-32211, Li-Cor, USA) for visualization. This work utilized the following antibodies: β-actin (Cat No. 66009-1-Ig, Proteintech) and PRDX1 (Cat No. 66820-1-Ig, Proteintech).

### 2.12 Cell counting kit-8

The Cell Counting Kit-8 (CCK8) was utilized to evaluate the proliferation of T24 and UMUC-3 cells. In accordance with the guidelines, cells (2 × 10³/well) were inoculated in 100 µL of media using 96-well plates, with three replicate plates established for various time points. CCK-8 reagent (AbMole BioScience) was applied at 10 µL per well at intervals of 0 to 96 hours. After a 2-hour incubation, absorbance was measured at 450 nm using a BioTek plate reader.

### 2.13 Colony formation assay

For clonogenic tests, 6-well plates were inoculated with 1 × 10³ cells per well in 2 mL of medium. Following an 8–10 days cultivation at 37°C with 5% CO_2_, colonies were fixed with 4% PFA (Biosharp #BL539A), stained with 0.1% crystal violet (Solarbio #G1063), and subsequently photographed and quantified.

### 2.14 Wound-healing assay

Transfected cells (6×10^5^) attained confluence 48 hours after transfection. Monolayers were scraped with sterile 200 μL tips, rinsed with PBS, and subsequently treated with in serum-free media. Migration was evaluated by photographing wounds at 0 and 24 hours using inverted microscopy, with closure rates measured using ImageJ. Transwell migration assay BLCA cells (1×10^5^ in 200 μL of serum-free media) were inoculated into LABSELECT chambers (8 μm holes; #14342). The lower chambers had 600 μL of RPMI-1640 enriched with 20% FBS as a chemoattractant. After 24–48 hours of incubation at 37 °C with 5% CO_2_, the transmigrated cells were subjected to methanol fixation (4%), crystal violet staining (0.1%; Solarbio #G1063), and subsequent microscopic counting.

### 2.15 Nile red staining and ROS assays

Lipid accumulation in PRDX1-silenced cells was visualized using Nile Red fluorescent staining (Solarbio, China). Cells were rinsed with PBS, fixed in 4% paraformaldehyde, and treated with 500 μL of Nile Red reagent for 15 minutes under light-protected conditions. To measure intracellular reactive oxygen species (ROS), BC cells cultured in six-well plates were exposed to 25 μM DCFH-DA probe (HY-D0940, MedChemExpress) and maintained in darkness for 30 minutes. Both lipid and ROS fluorescence signals were documented through confocal microscopy imaging.

### 2.16 Statistical analysis

R software (version 4.3.3) along with GraphPad Prism (version 9.0) were utilized for conducting statistical evaluations. Based on distribution patterns, continuous variables underwent analysis through either Student’s t-test or Wilcoxon rank-sum test. For categorical data, χ² test or Fisher’s exact test was applied as appropriate based on anticipated frequencies. Kaplan-Meier methodology was employed to analyze survival data, with log-rank test comparing groups, supplemented by Cox proportional hazards regression for multivariate analysis. Each experiment was replicated three times, with results presented as mean values accompanied by standard deviation (SD). A p-value below 0.05 indicated statistical significance. The following notation indicates significance thresholds: n.s. denotes non-significant results; *p ≤ 0.05; **p ≤ 0.01; ***p ≤ 0.001; ****p ≤ 0.0001.

## 3 Results

### 3.1 Single-cell data integration

**Figure 2:**
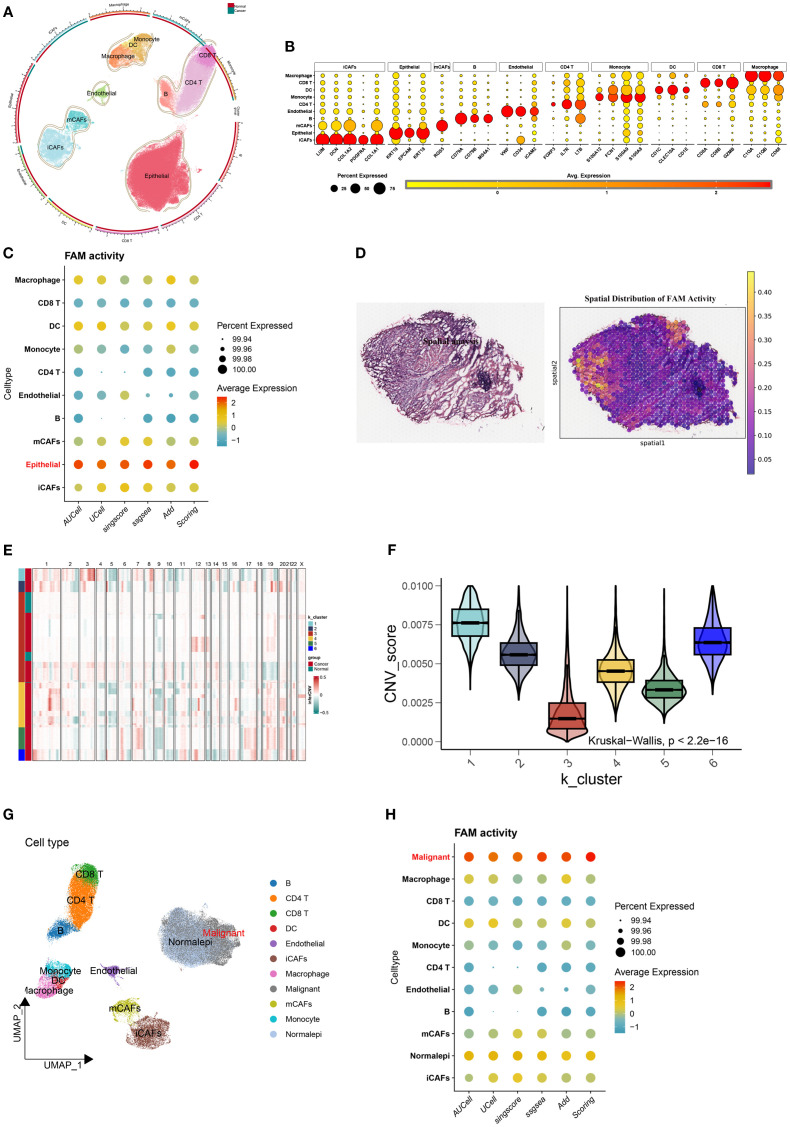
Elevated FAM activity in malignant epithelial cells of BLCA. **(A)** UMAP plot displaying the cellular landscape of BLCA. **(B)** Representative marker genes used to annotate each cell type. **(C)** Bubble plot showing FAM gene set enrichment scores across cell types, calculated using AUCell, UCell, singscore, ssGSEA, AddModuleScore, and Scoring. **(D)** H&E staining and corresponding heatmaps illustrating the spatial distribution of FAM activity. **(E)** Malignant cells identified through K-means clustering based on inferred CNV profiles. **(F)** CNV score differences among six clusters. **(G)** Refined cell annotation confirming the identity of malignant cell populations. **(H)** Bubble plot indicating higher FAM enrichment scores in malignant cells, consistently observed across multiple scoring algorithms.

Batch effect adjustment was conducted to combine seven single-cell datasets containing 34 specimens (4 control tissues and 30 tumor tissues) (**Supplementary Figure S1A-C**). Classical BLCA markers were employed to identify cellular subtypes, which included Macrophages, Monocytes, Dendritic Cells (DCs), Endothelial Cells, B Cells, CD4 T Cells, CD8 T Cells, Epithelial Cells, myofibroblast Cancer-Associated Fibroblasts (mCAFs), and inflammatory Cancer-Associated Fibroblasts (iCAFs) (**Figure 2A**). Marker gene bubble plots were generated to verify annotation precision (**Figure 2B**). Five distinct computational methods (AUCell, UCell, singscore, ssGSEA, and AddModuleScore) revealed markedly increased FAM activity within epithelial regions across all approaches (**Figure 2C**; **Supplementary Figure S2A-C**). Spatial transcriptomics profiling further revealed pronounced FAM signature enrichment within tumor core regions of BLCA specimens (**Figure 2D**). Given substantial heterogeneity among epithelial cells, we employed inferCNV to delineate malignant clones. This analysis identified five distinct CNV clusters, with Cluster 1 exhibiting the highest malignancy score (**Figures 2E, F**; **Supplementary Figure S2D**). Subsequent UMAP-based reannotation epithelial cells (**Figure 2G**), which demonstrated higher composite FAM activity than normal epithelial controls (**Figure 2H**; **Supplementary Figure S1E, F**).

### 3.2 Dissecting FAM in malignant BLCA cells

**Figure 3:**
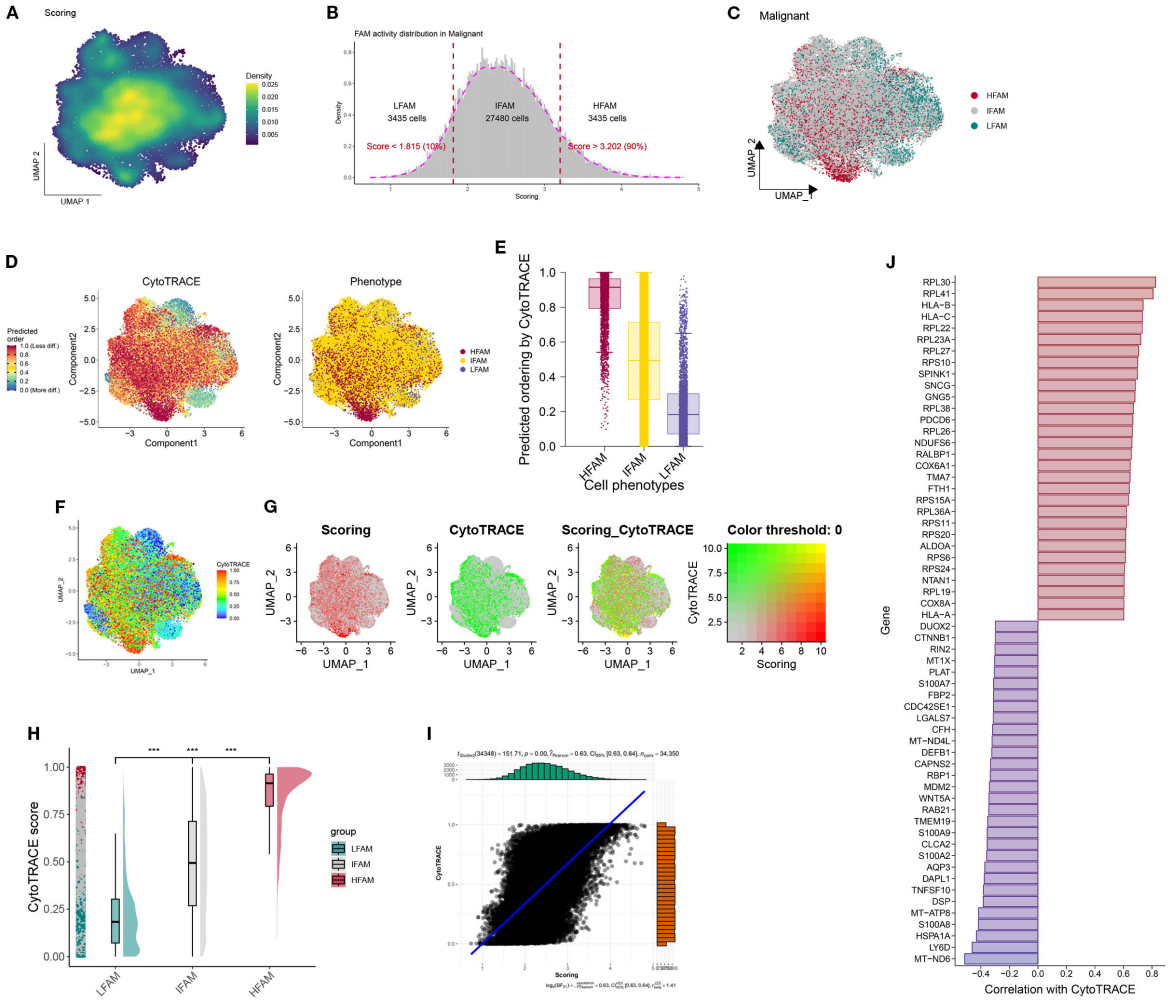
Identification and characterization of FAM-associated malignant cell subsets. **(A)** UMAP visualization showing the heterogeneity of FAM activity among malignant cells. **(B, C)** Malignant cells were stratified into three groups-LFS, DTFS, and HFS-based on FAM activity scores. **(D)** The CytoTRACE characteristics and FAM-related phenotypes of BLCA cells. **(E)** Boxplots showing differentiating ordering identically ordered by CytoTRACE. **(F–G)** The correlation was revealed when the FAM score was combined with the CytoTRACE score. **(H)** Comparison of CytoTRACE Scores Among LHS, LDTS and LLS. **(I)** Pearson correlation test for CytoTRACE and FAM scores **(J)** The expression Profiles of Cell-Specific Marker Genes Linked to CytoTRACE Analysis. ***P < 0.001.

The UMAP revealed heterogeneous FAM activity patterns within tumors, particularly evident in malignant epithelial populations (**Figure 3A**). Based on quartile distributions of FAM scores, we stratified cells into three distinct categories: LFS, DTFS, and HFS populations (**Figures 3B, C**). The differentiation status of cancer cells was further investigated using the Monocle2 algorithm through CytoTRACE analysis. The results demonstrated that LHS cancer cells exhibited a lower degree of differentiation and possessed a higher potential for differentiation (**Figures 3D, E**). We subsequently correlated the FAM score with the CytoTRACE score, as illustrated in **Figures 3F, G**. The yellow regions indicate cells that exhibit high FAM and CytoTRACE scores, which largely demonstrate a consistent overlap. We then conducted an analysis of the CytoTRACE scores across the three groups (**Figure 3H**). Additionally, our findings revealed a significant correlation between CytoTRACE and FAM scores (**Figures 3I**). **Figure 3J** illustrates the expression profiles of cell-specific marker genes in relation to CytoTRACE.

### 3.3 Functional profiling of FAM in scRNA-seq data

**Figure 4:**
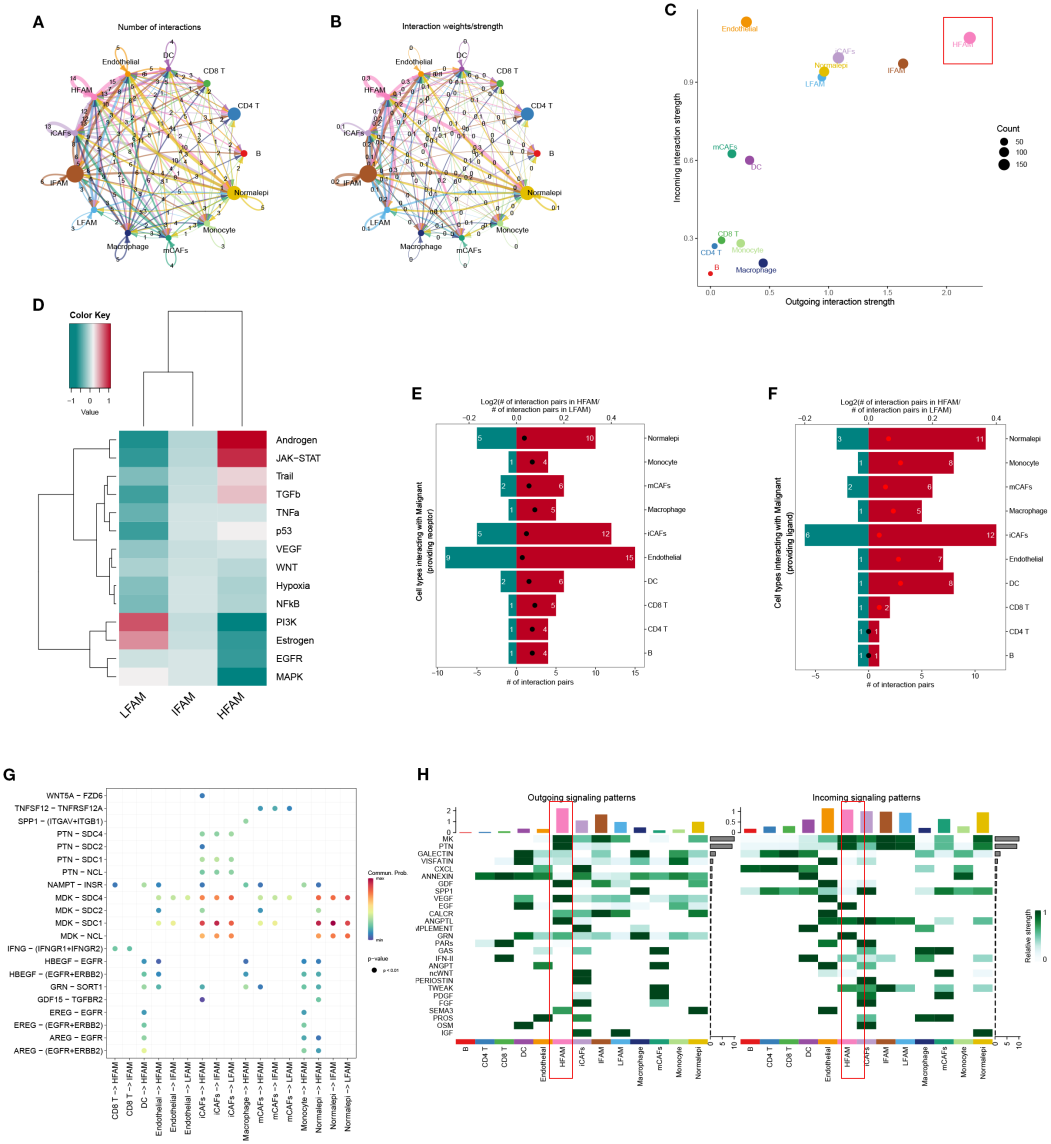
Functional characterization of HFS, DTFS, and LFS malignant cells based on scRNA-seq–derived cell–cell communication analysis. **(A, B)** CellChat analysis depicting interaction frequency and strength between malignant subgroups (HFS, DTFS, LFS) and other cell types. **(C)** Overview of intercellular communication patterns across various cell populations. **(D)** Heatmap showing differential enrichment of signaling pathways among HFS, DTFS, and LFS cells. **(E, F)** Bar plots illustrating the number of predicted interactions between HFS or LFS malignant cells and surrounding cell types. **(G)** Visualization of communication networks linking malignant subgroups with other cell populations. **(H)** Heatmap displaying the inferred probability of incoming and outgoing signaling events for each subgroup.

To investigate intercellular communication patterns, we applied CellChat to the scRNA-seq data and analyzed interactions between LFS, DTFS, HFS, and other cell populations. This analysis quantified both interaction frequency and signaling strength across cell types (**Figures 4A, B**). HFS cells demonstrated notably higher signaling activity, indicating superior intercellular communication capacity (**Figure 4C**). Further pathway enrichment analysis identified key signaling routes that were upregulated in HFS cells, including the Androgen and JAK-STAT pathways (**Figure 4D**). To refine the comparison between HFS and LFS cells, we assessed ligand–receptor pair expression. HFS cells showed a marked increase in potential communication events with neighboring cell types, as reflected by a greater number of ligand–receptor pairs (**Figures 4E–G**). Consistently, heatmap visualization revealed elevated interaction probabilities in the HFS subgroup (**Figure 4H**).

### 3.4 Revealing HFS-specific co-expression modules using hdWGCNA

**Figure 5:**
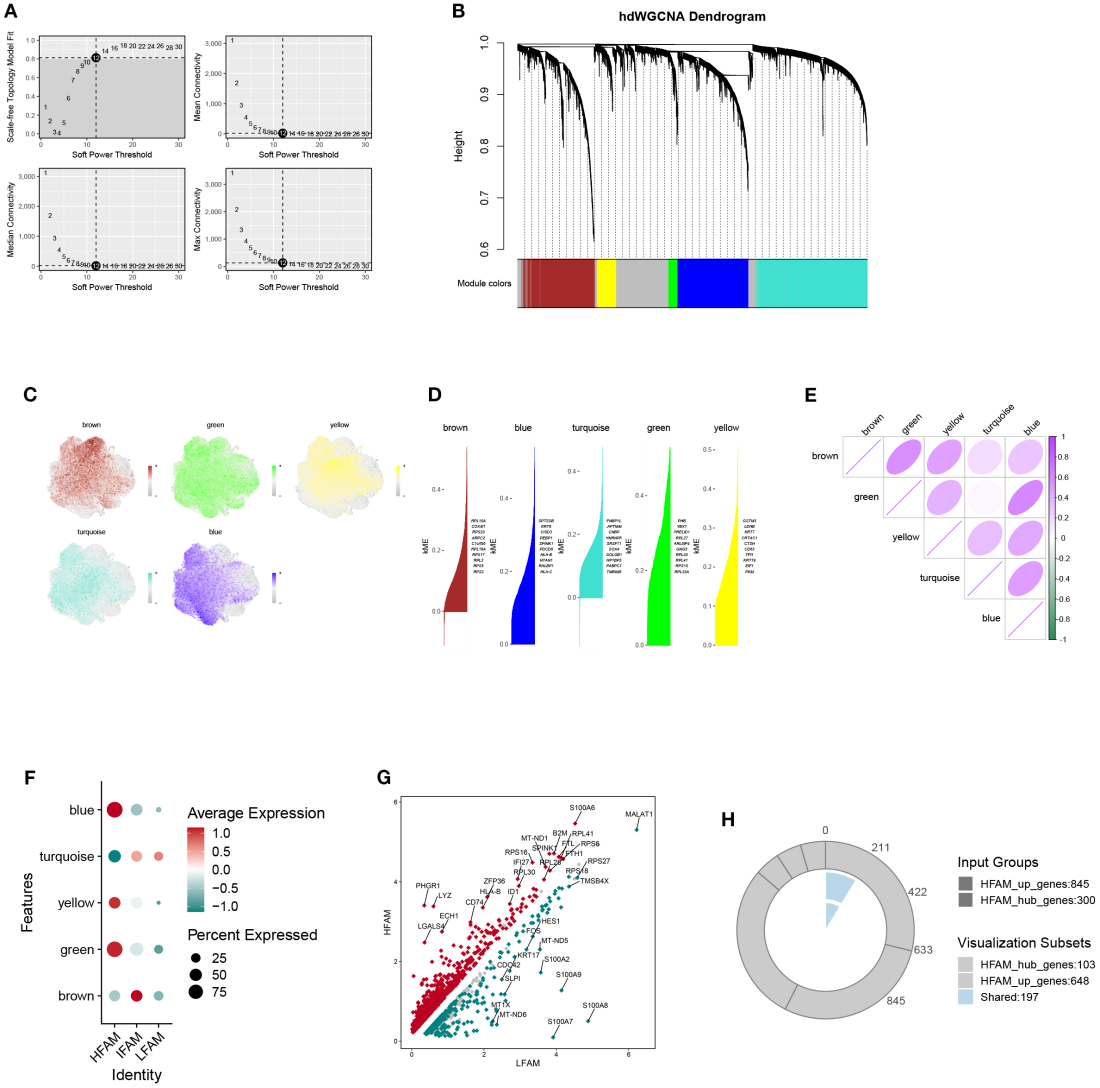
Identification of key gene modules associated with HFS malignant cells via hdWGCNA. **(A)** Plot of scale-free topology fit index and mean connectivity across a range of soft-thresholding powers. **(B)** Hierarchical clustering dendrogram showing gene module classification; five distinct modules were identified using the hdWGCNA framework. **(C)** UMAP visualization displaying the distribution of module feature scores across malignant cells. **(D)** Top hub genes identified within each module based on intramodular connectivity. **(E)** Heatmap illustrating correlation patterns among the five modules. **(F)** Bubble plot summarizing module scores for the five identified gene modules. **(G)** The volcanic map of the DEGs. **(H)** Venn diagram showing the overlap between module-derived genes and DEGs, identifying shared candidates for further analysis.

The hdWGCNA framework was employed to detect gene co-expression networks among malignant cells exhibiting high FAM scores. We established a scale-free topology using a soft-thresholding parameter set at 12, which led to the discovery of five separate modules (**Figures 5A, B**). The cellular distribution within each module was visualized through UMAP projection (**Figure 5C**). A heatmap further displayed the relationships between different modules (**Figures 5D, E**). Analysis via bubble plots demonstrated pronounced correlations of the blue, green, and yellow modules with cells displaying elevated FAM activity (**Figures 5F**). Subsequently, we extracted genes from these three key modules (blue, green, and yellow) using a module membership threshold (kME) exceeding 0.3, which generated 300 genes for downstream investigation (**Supplementary Table S4**). Comparing gene expression profiles of HFS versus LFS populations identified 1205 genes with increased expression linked to elevated FAM activity (**Supplementary Table S5**). cross-referencing these module-associated genes with the DEGs identified earlier produced 197 potential candidates involved in FAM enhancement within BLCA (**Figure 5M**; **Supplementary Table S6**).

### 3.5 Machine learning–based identification of OFGs

**Figure 6:**
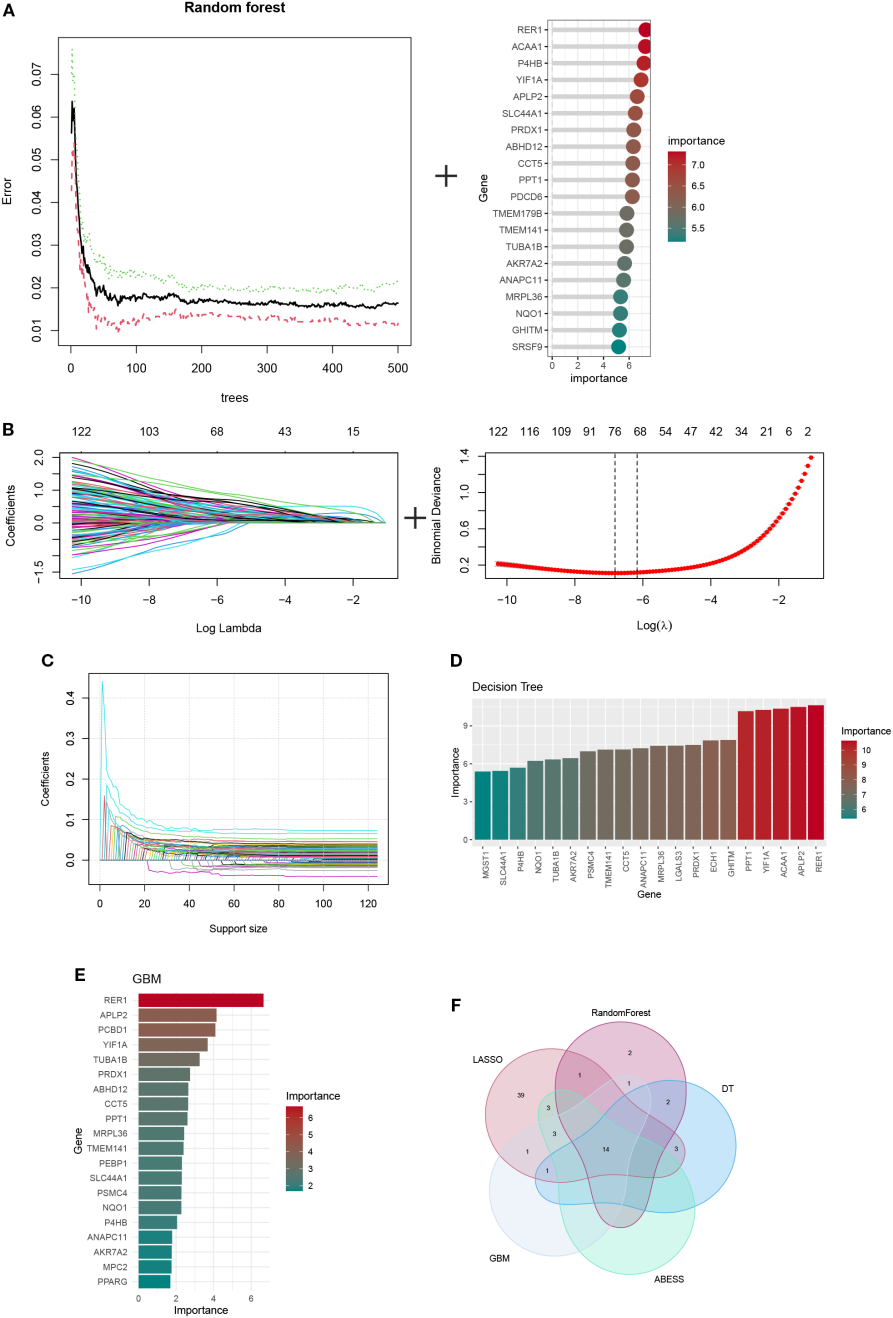
The identification of hub ORGs is performed using machine learning techniques, **(A)**RF algorithm **(B)** the LASSO regression algorithm. **(C)** Adaptive BEst Subset Selection (ABESS) algorithm. **(D)** Decision Tree (DT) algorithm. **(E)** Gradient Boosting Machine (GBM) algorithm. **(F)** Venn diagrams of five algorithms.

To identify the optimal feature genes (OFGs), multiple machine learning algorithms were applied for screening. Random Forest analysis identified a total of 166 candidate genes (**Figure 6A**), while LASSO regression yielded 65 genes (**Figure 6B**). ABESS further selected 20 genes (**Figure 6C**). Additionally, 20 genes were identified using DT and GBM methods (**Figures 6D, E**; **Supplementary Table S7**). By intersecting the gene sets obtained from these five algorithms, 14 hub genes were consistently shared across methods: TUBA1B, YIF1A, AKR7A2, NQO1, PRDX1, PPT1, ANAPC11, P4HB, CCT5, TMEM141, SLC44A1, MRPL36, APLP2 (**Supplementary Table S8**).

### 3.6 Single-cell resolution analysis of identified OFGs

**Figure 7:**
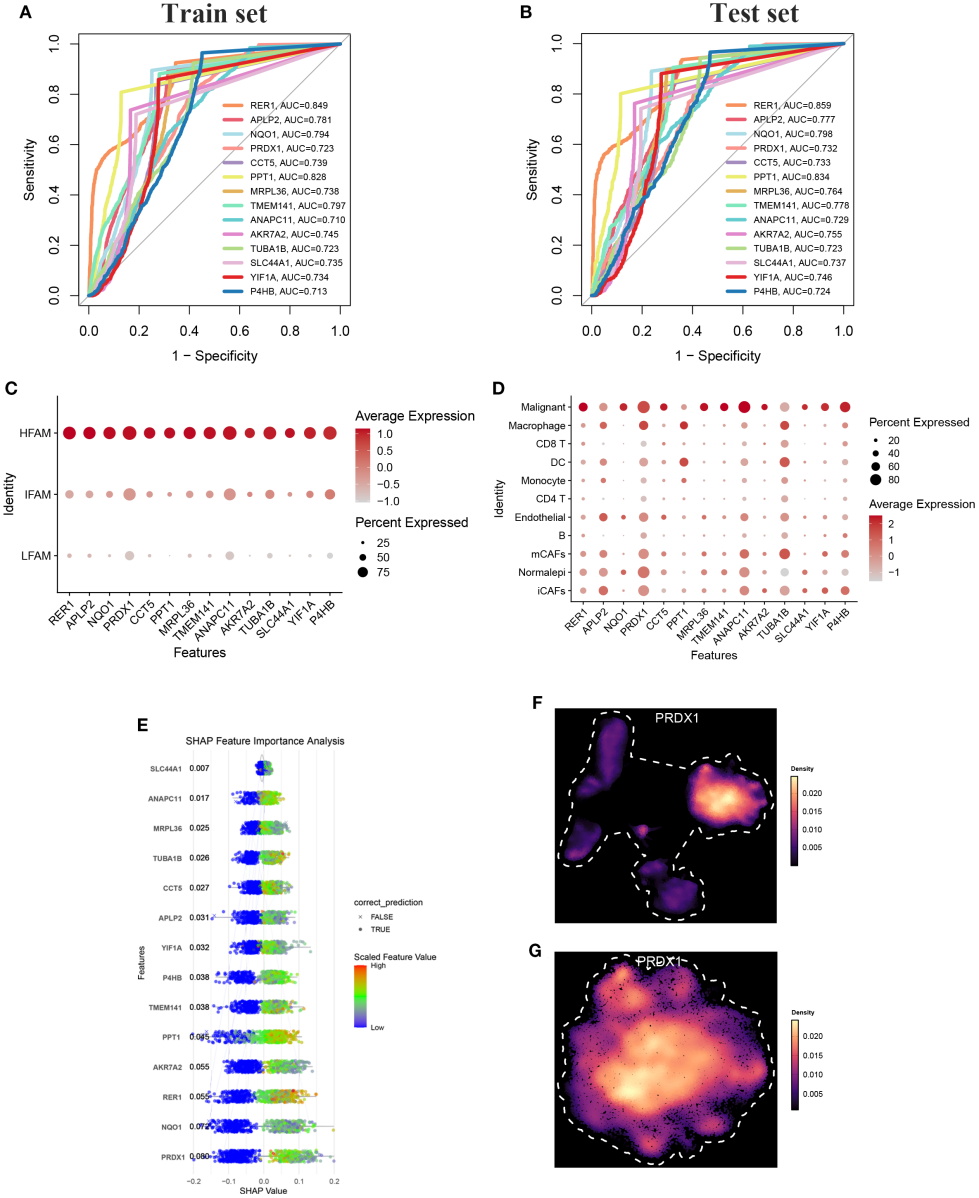
**(A)** ROC-AUC performance of top candidate biomarkers in the training cohort. **(B)** ROC-AUC performance of top candidate biomarkers in the testing cohort **(C, D)** The bubble chart shows the expression of all characteristic genes in HFAM and malignant cells. **(E)** SHAP summary plot showing feature importance across the predictive model. points shifted to the left indicate negative impact (protective effect), while points shifted to the right indicate positive impact (risk-enhancing effect) on model prediction. **(F, G)** UMAP analysis revealed that PRDX1 was highly expressed in epithelial cells and malignant tumor cells.

The HFS subgroup in the single-cell dataset was randomly divided into training and testing cohorts. Diagnostic performance of the identified OFGs remained robust in both sets, with all genes showing AUC values above 0.7 (**Figures 7A, B**). In the previously defined HFS, DTFS, and LFS groups, OFGs were significantly enriched in HFS cells, exhibiting a marked upward trend in expression levels (**Figure 7C**). Overall, these genes were predominantly expressed in malignant cells, which also showed the highest average expression (**Figure 7D**).

We performed 10 repetitions of fivefold cross-validation on the training set to assess the robustness of each model. Most models exhibited robustness in their predictions. Among these models, RPART performed poorly, while naïve Bayes demonstrated a balanced precision and sensitivity, showing an AUC of 0.992 (**Supplementary Figure S3A-C**). Therefore, the naïve Bayes algorithm was adopted in the final machine-learning model, SHAP analysis ranked the OFGs by their contribution to the predictive model, identifying PRDX1 as a key potential driver (**Figure 7E**). Notably, PRDX1 expression was consistently elevated across both the broader epithelial cell population (**Figure 7F**) and the malignant epithelial subset (**Figure 7G**).

### 3.7 Trajectory inference and signaling features of PRDX1^+^ malignant cells

**Figure 8:**
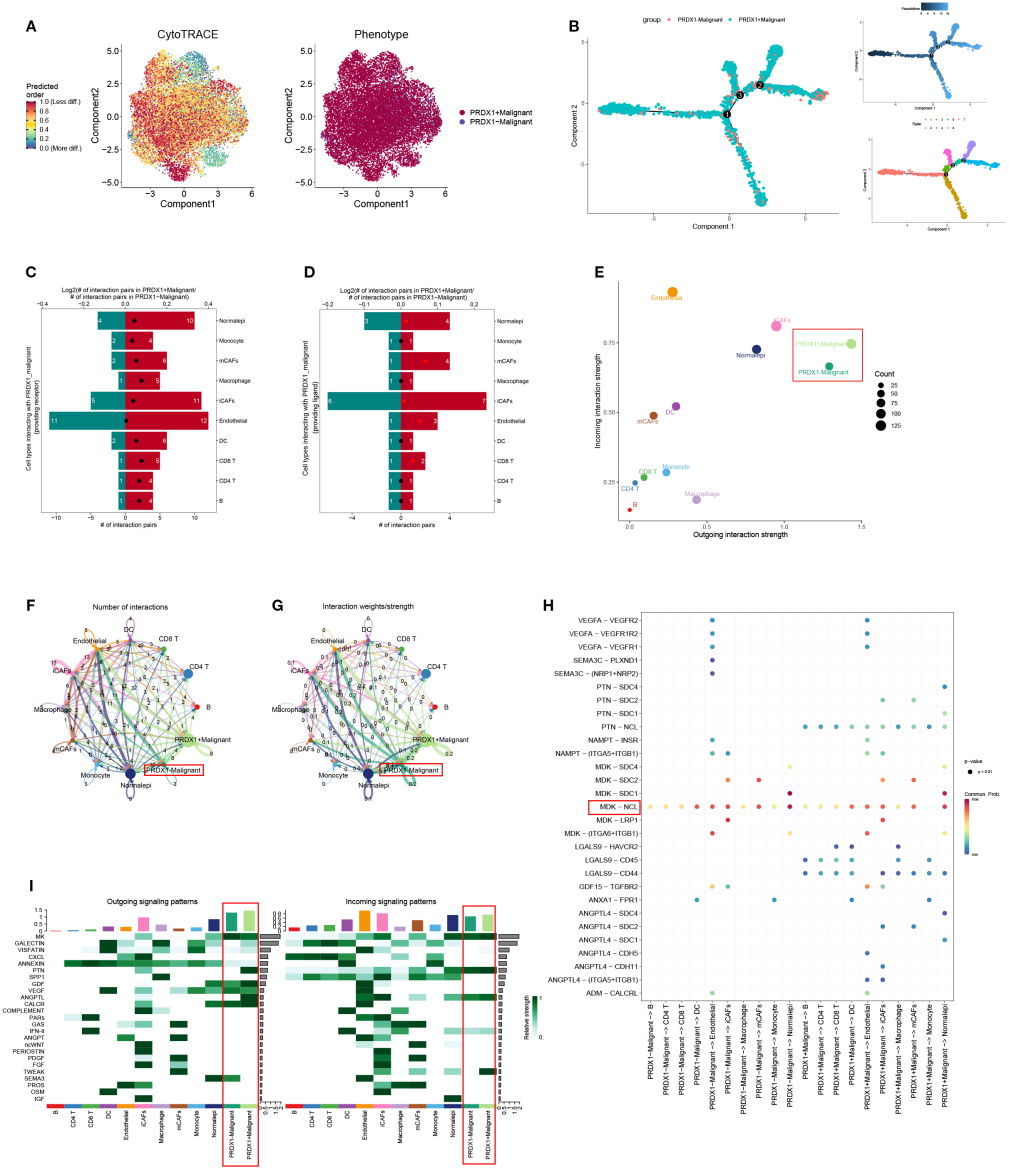
Trajectory analysis and cellular communication in PRDX1+ malignant cells. **(A)** The distribution of PRDX1 in malignant cells was visualized by a UMAP plot. **(B)** CytoTRACE analysis of PRDX1+ malignant cells. **(C)** Raincloud plot of CytoTRACE scores in PRDX1^+^ malignant cells and PRDX1 malignant cells. **(D, E)** Bar plots showed the number of interactions between PRDX1^+^ malignant cells and other cell types. **(F)** The correlation between differential outgoing contacts and the degree of incoming interactions in PRDX1^+^ malignant cells and PRDX1- malignant cells. **(G, H)** Quantity and intensity of cellular communications between PRDX1^+^ malignant cells and other cell types. **(I)** PRDX1^+^ malignant cells interacting with various cell ligand-receptor bubble diagrams. **(J)** A heat map summarizing the outgoing and incoming signal pathways of PRDX1^+^ malignant cells and other cell types.

To investigate the functional role of PRDX1, malignant cells from BLCA samples were stratified based on PRDX1 expression into PRDX1^+^ and PRDX1^-^ subsets. CytoTRACE analysis indicated that PRDX1^+^ cells exhibited significantly enhanced stemness features compared to their PRDX1^-^ counterparts (**Figure 8A**; **Supplementary Figure S4**). Pseudotime trajectory analysis revealed that PRDX1^+^ high malignant cells preferentially occupy a late-stage transcriptional state along Component 1, whereas PRDX1-low/negative cells map to earlier pseudotime positions, indicating that PRDX1 expression marks a progressive, more advanced malignant phenotype (**Figure 8B**). We comprehensively evaluated the intercellular communication profiles of PRDX1^+^ and PRDX1^-^ malignant cells (**Figures 8C–E**). PRDX1^+^ cells exhibited markedly increased numbers of ligand–receptor interactions and stronger signaling intensity with surrounding cell types, indicating globally enhanced communication capacity (**Figures 8F–G**). Intercellular communication analysis revealed that the MDK–NCL axis serves as a major route through which PRDX1^+^ malignant cells interact with diverse cell subtypes (**Figure 8H**). Moreover, PRDX1^+^ cells demonstrated elevated communication probabilities, as shown in the heatmap. GALECTIN, PTN, and ANGPTL pathways were significantly more active in PRDX1^+^ than in PRDX1^-^ cells. For incoming signals, TWEAK expression was predominantly enriched in PRDX1^+^ cells (**Figure 8I**).

### 3.8 Clinical relevance of PRDX1 in BLCA

**Figure 9:**
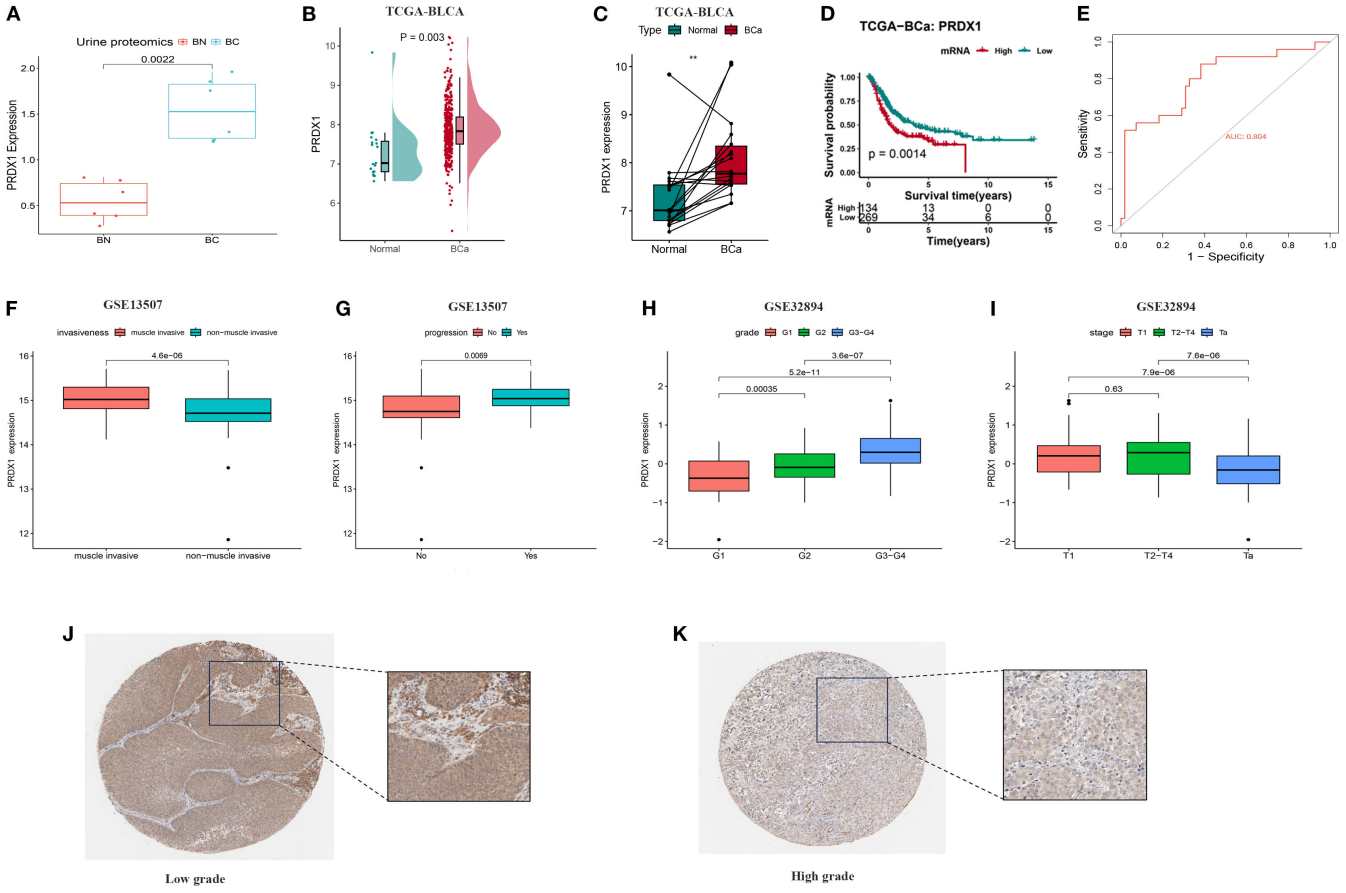
Differences in PRDX1 expression levels in BLCA and their correlation with clinical pathological features. **(A)** PRDX1 expression levels in urine proteomics; **(B)** PRDX1 expression levels in urine proteomics from 5 BLCA patients and 5 normal individuals; **(C)** Paired comparison of PRDX1 expression levels between 403 BLCA and 19 normal tissues; **(D)** Relationship between PRDX1 expression levels and overall survival of BC patients; **(E)** ROC curve assessing the sensitivity and specificity of PRDX1 as a predictive marker for BLCA. **(F)** PRDX1 expression in muscle-invasive vs. non–muscle-invasive bladder cancer samples in the GSE13507. **(G)** PRDX1 expression in bladder cancer patients with and without disease progression in GSE13507. **(H)** PRDX1 expression across tumor grades in the GSE32894 dataset. **(I)** PRDX1 expression across pathological stages in the GSE32894. **(J)** Immunohistochemical staining of PRDX1 in adjacent non-cancerous tissues; **(K)** Immunohistochemical staining of PRDX1 in BLCA tissues.

Previous proteomic profiling of urine samples from five individuals with BLCA and five healthy participants demonstrated substantial elevation of PRDX1 levels among cancer patients (**Figure 9A**), suggesting its value as a non-invasive diagnostic indicator for initial screening. The TCGA dataset provided additional confirmation, showing considerably higher PRDX1 levels in cancerous tissues relative to neighboring normal samples, which held true for matched tissue pairs (**Figures 9B, C**). Prognostic assessment revealed that reduced PRDX1 levels correlated with improved patient survival (**Figure 9D**). Additionally, ROC curve evaluation yielded strong diagnostic capability with an AUC value of 0.804 (**Figure 9E**). In bulk RNA-seq datasets, PRDX1 expression was notably elevated in muscle-invasive BLCA (**Figure 9F**) and showed a strong association with disease progression (**Figure 9G**), as well as with advanced tumor grade and stage (**Figures 9H, I**). Immunohistochemistry results obtained from the Human Protein Atlas (HPA) database further illustrated the spatial expression patterns of PRDX1 in tissue samples, corroborating its upregulation in tumor tissues (**Figures 9J, K**).

### 3.9 *In vitro* verification

**Figure 10:**
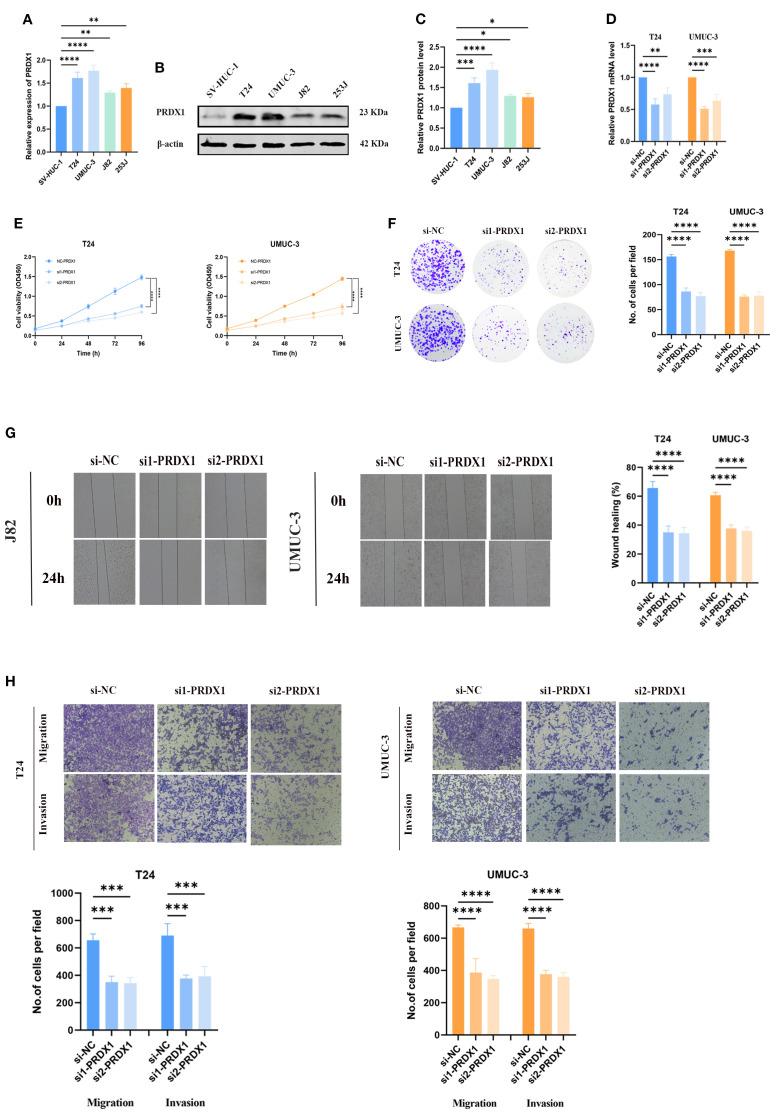
*In vitro* experiments of PRDX1. **(A)** qRT-PCR analysis showing the relative expression levels of PRDX1 in five cell lines (SV, UMUC-3, J82, T24, and 5637). **(B, C)** Western blot analysis demonstrating the expression of PRDX1 protein in these cell lines. **(D)** Determination of PRDX1 knockdown efficiency in T24 and UMUC-3 cells by qRT- PCR. **(E)** CCK-8 proliferation assay. **(F)** Colony formation experiment. **(G)** Wound-healing assay. **(H)** Trans-well migration assay. * p < 0.05, ** p < 0.01, *** p < 0.001, **** p < 0.0001.

To clarify the role of PRDX1 in BLCA, its expression was assessed across cell lines. Both RT-PCR and Western blot confirmed elevated PRDX1 levels in BLCA cells (T24, UMUC-3, J82, 253J) relative to normal SV-HUC-1 cells (**Figures 10A-C**). Effective knockdown in UMUC-3 and T24 was validated by qRT-PCR (**Figure 10D**). Functional assays demonstrated that PRDX1 silencing reduced proliferation (CCK-8, **Figure 10E**), colony formation (**Figure 10F**), and significantly impaired migratory and invasive capacities (wound healing and Transwell, **Figures 10G, H**). Our results indicated that knockout of PRDX1 resulted in a substantial increase in ROS levels (**Supplementary Figure S5A**) in addition, Nile Red staining results demonstrated a substantial reduction in intracellular lipid droplets following PRDX1 knockdown (**Supplementary Figure S5B**).

## 4 Discussion

Cancer cells characteristically undergo metabolic rewiring, which allows tumors to survive and flourish in challenging environments (30). Growing research indicates that disrupted FAM serves as a key factor driving BLCA progression, complementing recognized changes in glucose utilization and amino acid processing, including glutamine addiction (31). Although FAM deregulation occurs across various cancer types—such as breast, prostate, lung, and kidney tumors—the regulatory mechanisms and functional relevance within BLCA are still inadequately characterized (32).

Consistent with prior reports, we confirmed that PRDX1 is upregulated and associated with poor prognosis in BLCA. The unique contribution of our study lies in contextualizing PRDX1 within fatty acid metabolism heterogeneity, revealing its close association with lipid metabolic pathways in addition to its well-known antioxidant activity. This dual connection suggests a BLCA–specific role for PRDX1, expanding our understanding of its oncogenic functions.

We utilized scRNA-seq technology to profile FAM heterogeneity throughout bladder tumors. Applying five distinct scoring approaches to an assembled FAM gene panel revealed substantially increased FAM activity within epithelial cells, particularly those with malignant characteristics. Metabolic diversity was additionally validated through spatial transcriptomic analysis. Notably, FAM activity showed variation both between different cell populations and among malignant epithelial subsets. Activity scores enabled stratification of malignant cells into three categories: LFS, DTFS, and HFS populations. Our functional investigations revealed that HFS cells exhibited enhanced cell-cell communication capabilities and increased stemness characteristics.

We investigated the underlying molecular mechanisms by combining differentially expressed genes distinguishing HFS from LFS populations with central genes determined through hdWGCNA analysis, creating a preliminary FAM-related gene panel. Various machine learning techniques subsequently refined this panel to pinpoint critical markers associated with tumor heterogeneity and patient outcomes. Through this strategy, we discovered 14 OFGs linked to elevated FAM activity. By incorporating urine proteomic profiles, PRDX1 was identified as a pivotal biomarker, demonstrating robust predictive capacity, increased tumor expression, and correlation with unfavorable outcomes.

PRDX1 belongs to the peroxiredoxin protein family and participates in maintaining cellular redox balance while shielding cells against oxidative injury (33). Nevertheless, within tumors, such protective antioxidant activity might counterintuitively promote cancer cell persistence through ROS neutralization, thus enabling continued growth and apoptotic evasion (34). We observed markedly increased PRDX1 levels in both BLCA tissues and cultured cell lines, where elevated expression linked to worse patient outcomes and more aggressive clinical characteristics, including higher tumor grades and stages. In addition to its diagnostic and prognostic value, PRDX1^+^ malignant cells were found to exhibit enhanced stemness, increased intercellular communication, and enrichment of key oncogenic pathways, suggesting a potential role in maintaining aggressive tumor phenotypes. Previous studies in other cancers, including breast and lung cancer, have implicated PRDX1 in promoting epithelial–mesenchymal transition (EMT), immune evasion, and chemotherapy resistance (35). As a key antioxidant enzyme, PRDX1 is sensitive to overoxidation at its catalytic cysteine in response to various stress stimuli. In the nucleus, oligomeric PRDX1 interacts with transcription factors such as p53, c-Myc, NF-κB, and AR, thereby modulating gene expression involved in cell survival and death (36, 37). In the cytoplasm, PRDX1 exhibits anti-apoptotic activity by regulating ROS-dependent signaling pathways through effectors including ASK1, p66Shc, GSTpi/JNK, and c-Abl (38). Through these mechanisms, PRDX1 functions as a multifaceted regulator of cell growth, apoptosis, and differentiation. Increasing evidence indicates that PRDX1 and its redox-associated pathways contribute to tumor progression and metastasis in multiple cancers, including breast, lung, and esophageal malignancies (35). Recent studies have demonstrated that the antioxidant protein PRDX1 plays a pivotal role in sustaining lipophagic flux in macrophages. Loss of PRDX1 results in elevated oxidative stress, impaired autophagic processing, intracellular cholesterol accumulation, and decreased levels of free cholesterol. These disruptions lead to the suppression of the nuclear receptor NR1H3 activity, ultimately impairing cholesterol efflux and accelerating the development of atherosclerotic lesions (39). Although this mechanism has been primarily described in macrophages, it suggests a broader regulatory role for PRDX1 in lipid metabolism and cholesterol homeostasis, which may be relevant in the context of tumor-associated metabolic reprogramming.

In our urinary proteomics analysis, PRDX1 was found to be significantly upregulated in the urine of BLCA patients compared to healthy controls, suggesting its potential utility as a non-invasive diagnostic biomarker. This finding aligns with the growing interest in urine-based markers for BLCA, given the limitations and invasiveness of repeated cystoscopy in routine surveillance. Previous studies support our observation, Qun et al. identified PRDX1 is positively correlated with the recurrence and progression of BLCA in patients (40). Tabaei et al. compared protein profiles from NMIBC and MIBC tissue samples using two–dimensional electrophoresis followed by LC–MS/MS identification. This approach highlighted several potential non–invasive diagnostic and prognostic biomarkers, with PRDX1 showing a pronounced up–regulation in NMIBC (41). Whether similar mechanisms operate in BLCA warrants further investigation. Together, our results highlight PRDX1 as both a marker of malignancy and a potential therapeutic target in BLCA.

Recent studies have further revealed that PRDX1 not only functions as an antioxidant enzyme but also plays a critical role in regulating ferroptosis and lipid ROS metabolism. For instance, PRDX1 suppresses ferroptotic cell death by scavenging lipid peroxides, thereby promoting tumor cell survival under oxidative stress. In addition, PRDX1 has been implicated in modulating the tumor immune microenvironment, facilitating immune escape through redox-dependent pathways. These findings provide new mechanistic insight into our observation of PRDX1 upregulation in BLCA and suggest that targeting PRDX1 may represent a promising therapeutic strategy by sensitizing tumor cells to ferroptosis and restoring anti-tumor immunity.

Our analyses showed a consistent and robust correlation between PRDX1 expression and FAM activity, suggesting that PRDX1 may be involved in metabolic heterogeneity in BLCA. However, whether PRDX1 directly regulates FAM pathways in BLCA cells remains to be determined. The current evidence supports an association, but causality cannot be inferred. It is possible that PRDX1 upregulation represents part of a broader metabolic and oxidative stress adaptation program rather than a direct driver of lipid metabolism. Future mechanistic studies, such as lipidomics profiling in PRDX1 knockdown cells or CRISPR-based perturbation combined with metabolic flux assays, will be needed to clarify this relationship.

This study has several limitations. First, our functional validation was limited to *in vitro* assays, and *in vivo* studies such as xenograft models will be required to confirm the role of PRDX1 in tumor progression and therapeutic targeting. Second, the clinical validation relied on retrospective datasets; independent, prospective cohorts are needed to establish the robustness of PRDX1 as a biomarker. These limitations highlight the need for further experimental and translational studies to strengthen our findings.

## 5 Conclusion

This study provides the first single-cell–level characterization of FAM heterogeneity in BLCA and redefines a FAM-related gene signature. Integrating bulk RNA-seq and machine learning, we identified key FAM-associated genes, with PRDX1 validated as a central biomarker. These findings offer a foundation for personalized metabolic-targeted therapies in BLCA.

## Data Availability

The original contributions presented in the study are included in the article/**Supplementary Material**. Further inquiries can be directed to the corresponding authors.
