# Supplementary material for: Multi-omics dissection of fatty acid metabolism heterogeneity identifies PRDX1 as a prognostic marker in bladder cancer
Source: Front Immunol. 2025 Sep 11;16:1669822. doi: 10.3389/fimmu.2025.1669822 (PMC12460412; doi:10.3389/fimmu.2025.1669822)
Supplement: Supplementary file 1 [file Table1.docx]

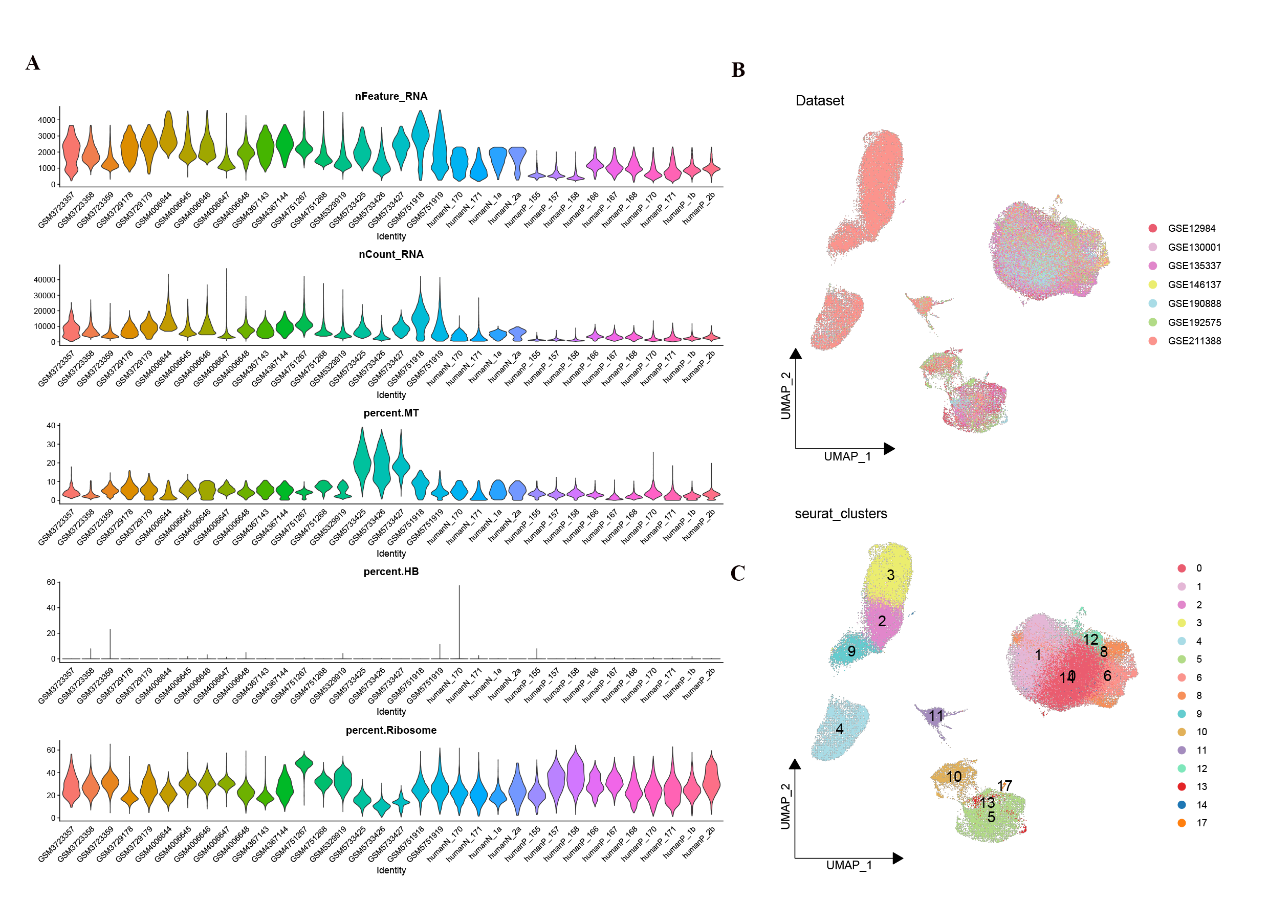


**Figure S1**

Filtering and processing of scRNA-seq data. (A) Quality control for inclusive data. (B) The cell distribution of the samples showed no significant batch effect. (C) The results of UMAP plot indicated that all cells were finely classified into 15 clusters. (D) Representative marker genes for each cell type.


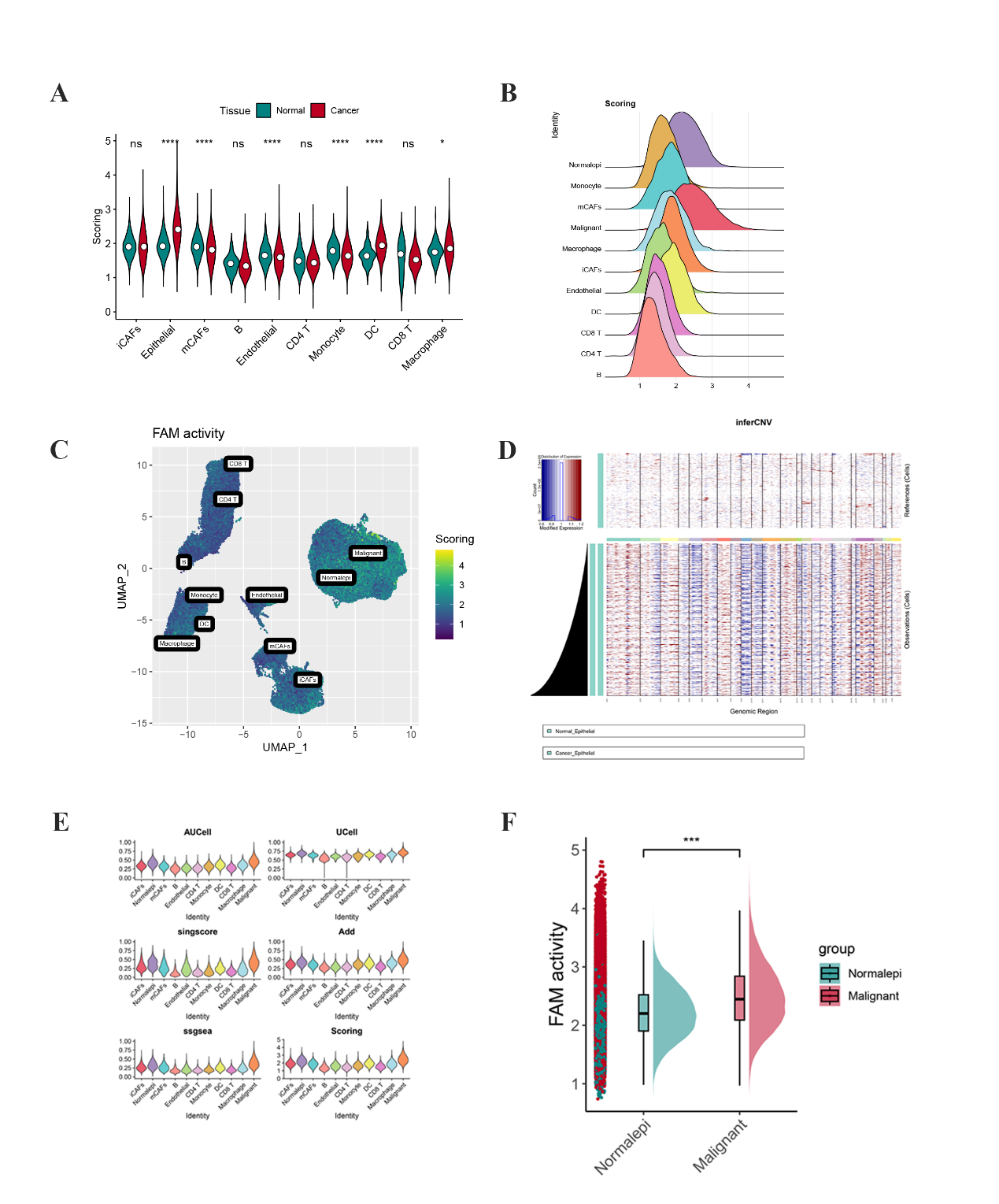


**Figure S2**

Heterogeneity among the expression of FAM gene set. (A-B) Violin plot and RidgePlot map showed expression scores of FAM activity for each cell type using AUCell, UCell, singscore, ssGSEA and Add algorithms. (C) UMAP plot showed the activity of FAM. (D) Graded heatmap showing CNVs of epithelial cells from tissues of each origin. (E-F) The results of AUCell, Ucell, singscore, ssGSEA and AddModuleScore algorithms showed that malignant cells had the highest aggregation activity.


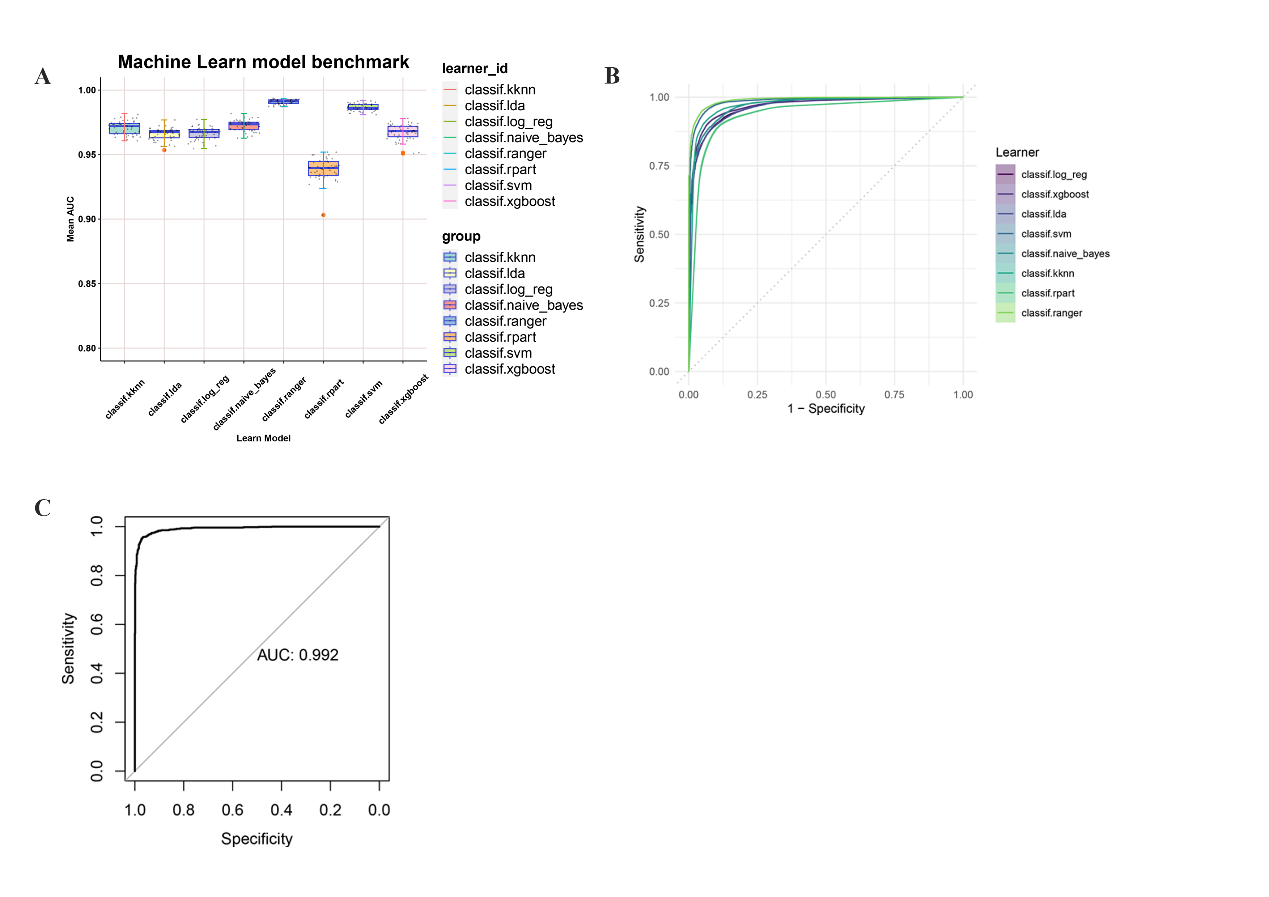


**Figure S3.** Benchmarking and performance of machine learning models.
(A) Comparison of classification performance across eight machine learning algorithms, including k-nearest neighbors (kNN), linear discriminant analysis (LDA), logistic regression, naïve Bayes, random forest (ranger), decision tree (rpart), support vector machine (SVM), and extreme gradient boosting (XGBoost). Model performance is shown as mean AUC values across resampling iterations.
(B) Receiver operating characteristic (ROC) curves for the different models, illustrating sensitivity versus 1–specificity.
(C) ROC curve of the final selected model, showing excellent performance with an area under the curve (AUC) of 0.992.


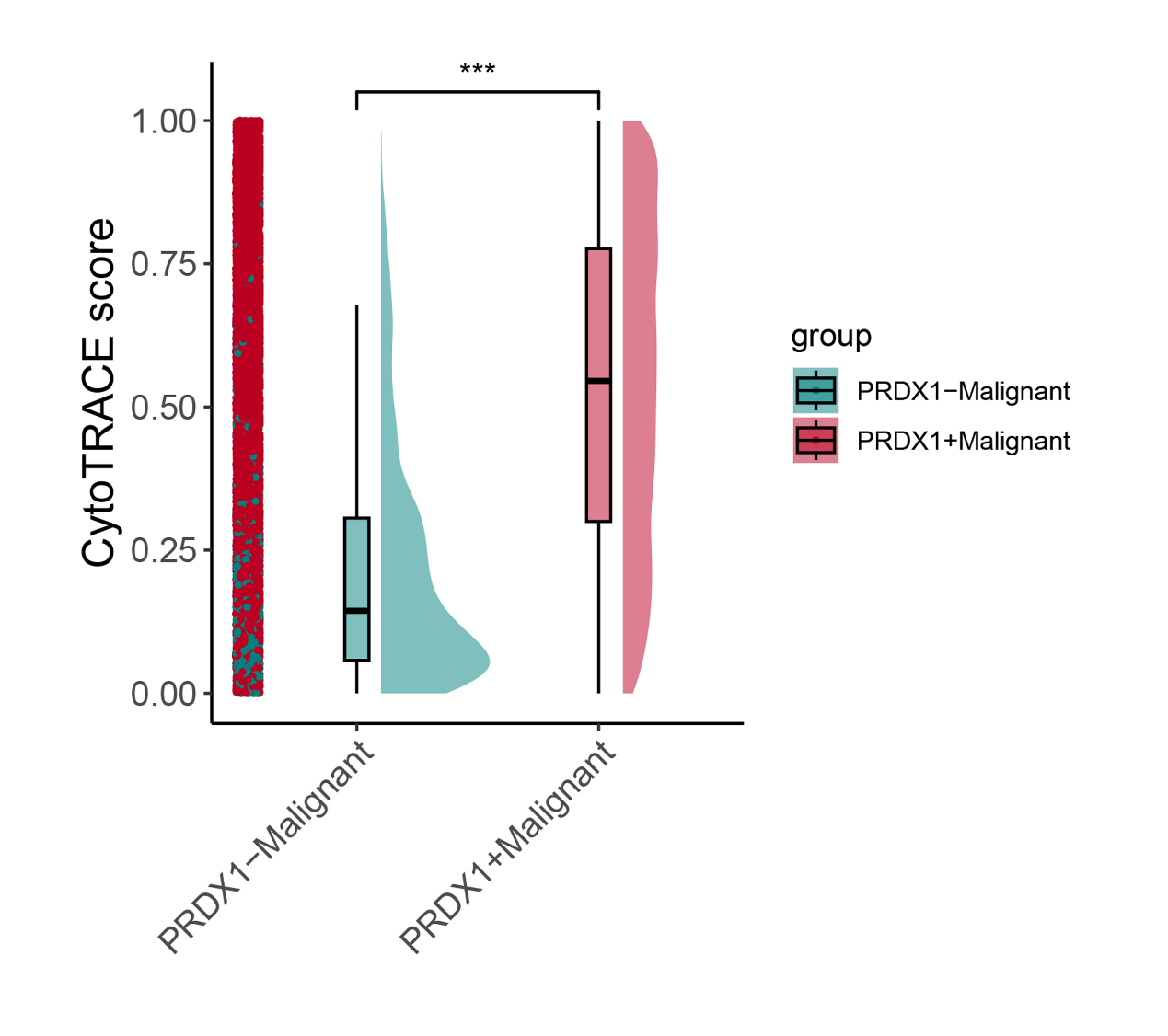


**Figure S4.** Raincloud plot of CytoTRACE scores in PRDX1+ malignant cells and PRDX1- malignant cells.


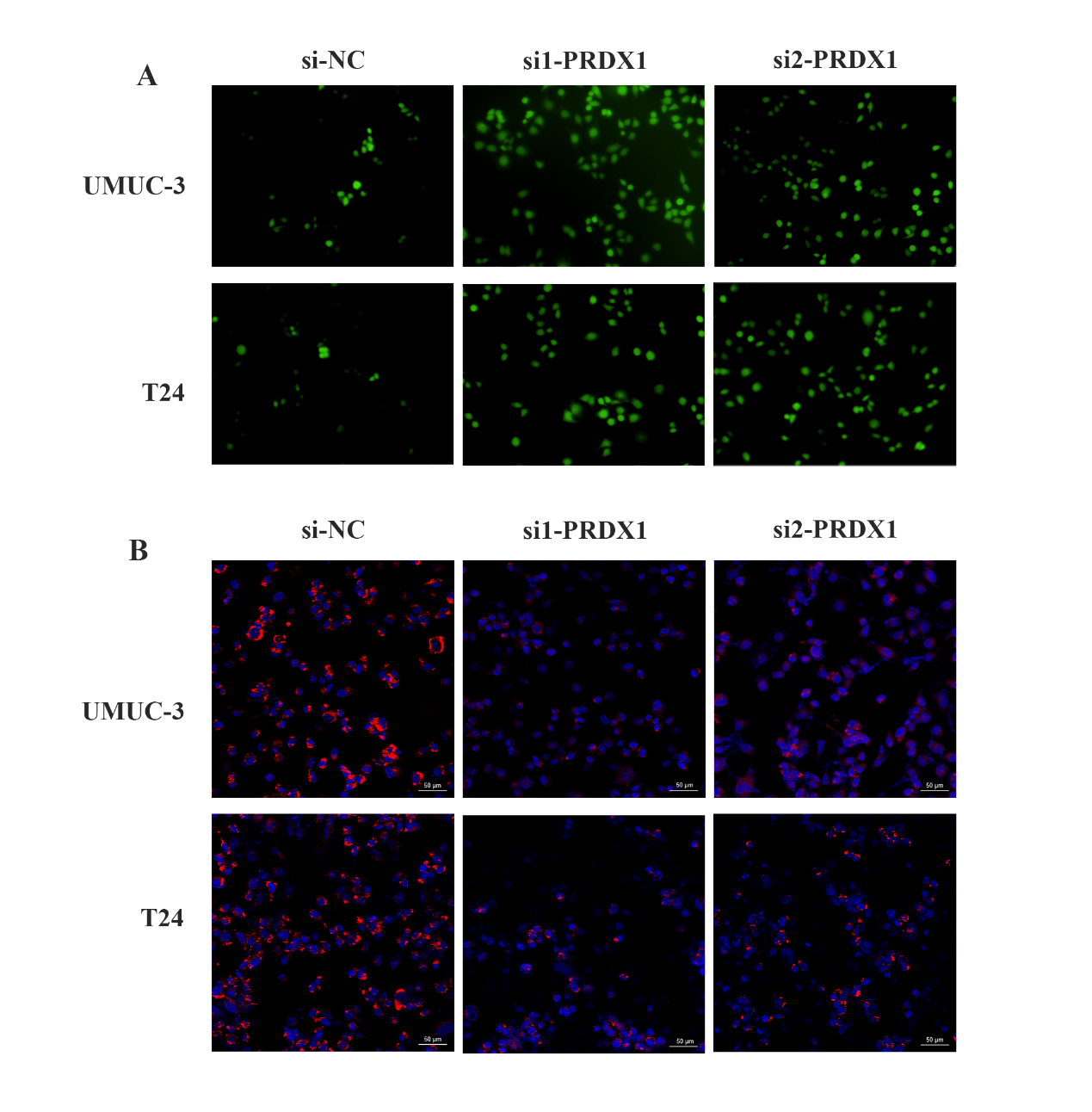


**Figure S5**. Impact of PRDX1 expression levels on ROS and Nile Red staining levels in BLCA cell lines. (A) Comparison of ROS fluorescence levels between si-PRDX1 cell lines and control groups; (B) Nile Red staining of BLCA cells following PRDX1 knockdown (Scale bar: 100 mm).
